# *Cistanche deserticola* Polysaccharide Reduces Inflammation and Aging Phenotypes in the Dermal Fibroblasts through the Activation of the NRF2/HO-1 Pathway

**DOI:** 10.3390/ijms242115704

**Published:** 2023-10-28

**Authors:** Kento Takaya, Toru Asou, Kazuo Kishi

**Affiliations:** Department of Plastic and Reconstructive Surgery, Keio University School of Medicine, Shinjuku, Tokyo 160-8582, Japan

**Keywords:** *Cistanche desserticola*, SASP, fibroblasts, skin aging, rejuvenation

## Abstract

Dermal fibroblasts maintain the skin homeostasis by interacting with the epidermis and extracellular matrix. Their senescence contributes to functional defects in the skin related to aging. Therefore, there is an urgent need for novel therapeutic agents that could inhibit fibroblast senescence. In this study, we investigated the effects of *Cistanche deserticola* polysaccharide (CDP), a natural anti-inflammatory component, on the progression of senescence in human dermal fibroblasts. Normal human dermal fibroblasts (NHDFs) were cultured in passages, and highly senescent cells were selected as senescent cells. CDP treatment increased the cell proliferation in senescent NHDFs and decreased the proportion of senescence-associated-β-galactosidase-positive cells. The treatment suppressed the senescence-related secretory phenotype, and reactive oxygen species (ROS) production was reduced, alleviating H_2_O_2_-induced oxidative stress. CDP mitigated ROS formation via the nuclear factor erythroid 2-related factor/heme oxygenase-1 pathway in senescent cells and was involved in the suppression of upstream p-extracellular signal-regulated kinase. These results indicate that CDP is an antioxidant that can alleviate age-related inflammation and may be a useful compound for skin anti-aging.

## 1. Introduction

Skin aging, similar to the aging of other organs, is characterized by a gradual loss of function and regenerative capacity. It is a multifactorial process that affects almost every biological and functional aspect of the skin. The skin is a mechanically protective and flexible barrier organ, and any changes, including aging, are very prominent and visible; therefore, it is important to control these changes to improve the quality of life in old age [1]. Skin aging is caused by both endogenous and exogenous factors. The endogenous factors that cause skin aging include time, genetic factors, and hormones, whereas the exogenous factors include sunlight, air pollution, cigarette smoke, nutritional factors, temperature, stress, and lack of sleep [2,3]. Both are oxidative processes associated with a progressive decline in the antioxidant capacity and increased reactive oxygen species (ROS) generation with aging [4]. The clinical signs of inherent skin aging are fine lines, dry skin, and laxity [5]. The aging process itself includes a variety of interdependent features at the molecular, cellular, and organ levels; however, the accumulation of senescent cells in the tissues is thought to contribute to aging.

There is growing evidence that suggests that senescent cells accumulate not only in the aged skin but also in prematurely aged skin and may contribute to age-related skin changes and disease states. Fibroblasts accumulate in the skin, particularly in the dermis and subcutaneous fat depots, and produce cytokines, extracellular matrix-modifying enzymes, and other molecules that can act remotely despite permanent mitotic arrest and the senescence-associated secretory phenotype (SASP), which may have far-reaching effects on the microenvironment of the adjacent cells [6,7,8]. Both endogenous and exogenous factors may induce the permanent aging of skin cells by shortening the telomeres, causing mitochondrial damage and upward regulation of DNA damage response signaling, ultimately leading to cell-cycle arrest with preserved metabolic activity [9,10]. Senescent fibroblasts are characterized by decreased papillary phenotype and increased reticular phenotype in the skin, and alpha smooth muscle actin (SMA) positivity [11,12]. Indeed, in a culture model of fibroblasts in the dermis, the number of cells exhibiting a reticular phenotype and SMA-positive myofibroblasts increased with increasing numbers of normal human dermal fibroblast (NHDF) passages [13]. It is believed that aging fibroblasts contribute to decreased skin integrity and function [14]. Therefore, improvement in aging-associated skin function requires removal of senescent fibroblasts or SASP suppression. Various studies have reported that control of senescent cells in the skin using drug-based approaches improved the aging phenotype [15,16,17]. However, some of these drugs had serious side effects [18], and people are increasingly seeking natural and safer antioxidant and anti-aging drugs. Natural antioxidants are being used widely in functional foods, cosmetics, and pharmaceuticals.

*Cistanche deserticola*, known as “desert ginseng,” contains natural anti-inflammatory, antifatigue, and anti-tumor ingredients [19,20,21]. *Cistanche deserticola* polysaccharide (CDP) is one of its major components. It has antioxidant and hepatoprotective properties and has been reported to protect the cells from oxidative stress (OS) damage in oxygen and glucose deprivation/reperfusion and osteoporotic conditions [22,23]. It induces melanogenesis in melanocytes and reduces OS by activating the nuclear factor erythroid 2-related factor (Nrf2)/heme oxygenase-1 (HO-1) pathway [24]. However, its effects on aging dermal fibroblasts, the largest constituent cells of the skin, are unknown.

Thus, we investigated the anti-aging and antioxidant effects of CDP on aging fibroblasts.

## 2. Results

### 2.1. Protective Effects of CDPs in Senescent Normal Human Dermal Fibroblasts (NHDFs)

**Figure 1:**
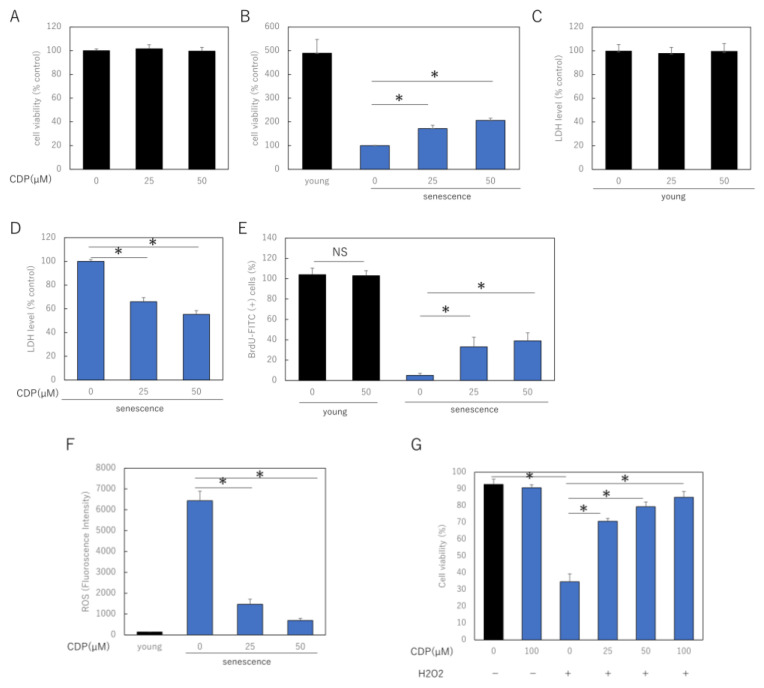
Effect of CDP on NHDF viability, injury, and proliferative activity. (**A**,**B**) Effect of CDP on cell viability in (**A**) young and (**B**) senescent NHDFs; (**C**,**D**) effect of CDP on cellular LDH release in (**C**) young and (**D**) senescent NHDF; (**E**) effect of CDP on the proliferative activity of senescent NHDF; (**F**) effect of CDP on ROS generation in aged NHDF; (**G**) protective effects of CDP against H_2_O_2_-induced oxidative stress. * *p* < 0.05.

To investigate the effect of CDP on skin aging, we treated young and senescent NHDF with CDP and determined the cell viability. After 72 h of treatment with CDP (25 μM and 50 μM), cell viability was significantly increased in highly senescent NHDF, although mitotic potential was reduced in the senescent cells. However, this treatment did not affect young low-successional NHDF (Figure 1A,B). In senescent NHDFs, cell damage during normal culture, measured using lactate dehydrogenase (LDH) release, was reduced by CDP treatment; however, the treatment did not affect LDH release in young NHDFs (Figure 1C,D). Furthermore, BrdU assay, which evaluates proliferative capacity, showed a significant improvement in proliferative capacity in CDP-treated senescent NHDFs, while the proliferative capacity of young cells was unaffected (Figure 1E). CDP treatment significantly decreased ROS production in senescent NHDFs (Figure 1F).

We further investigated the role of CDP on OS-induced stress damage NHDF using H_2_O_2_. To examine the effect of CDP on H_2_O_2_-induced cytotoxicity, NHDF cells were pretreated with CDP at various concentrations (25, 50, and 100 μg/mL) for 24 h, followed by H_2_O_2_ treatment. H_2_O_2_ treatment reduced the cell viability, whereas CDP significantly ameliorated this detrimental effect (Figure 1G).

### 2.2. Anti-Aging Effects of CDP Treatment in Senescent NHDFs

**Figure 2:**
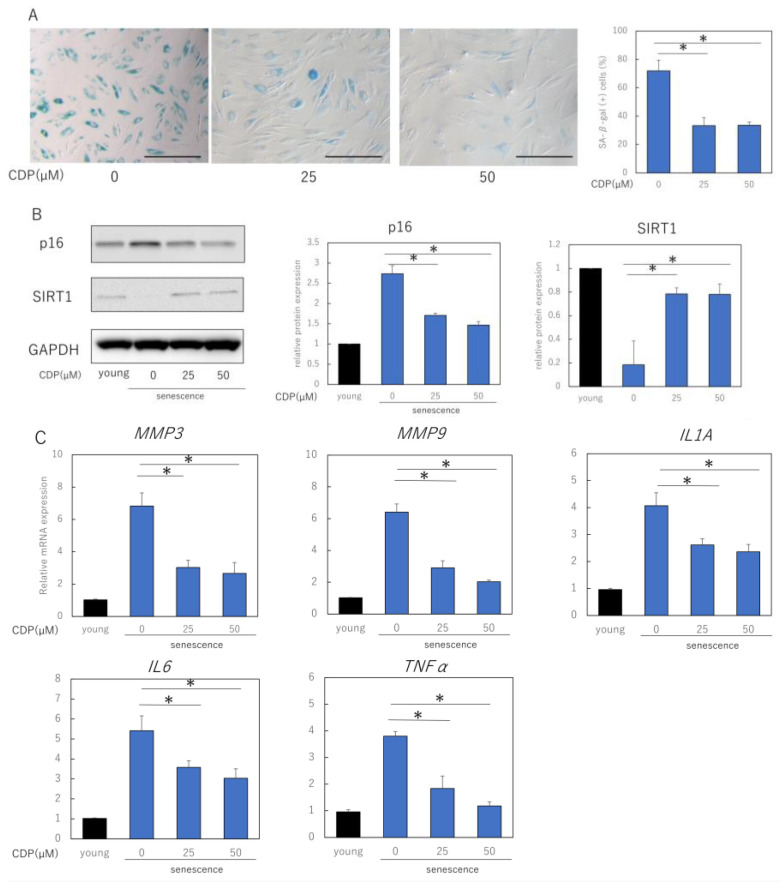

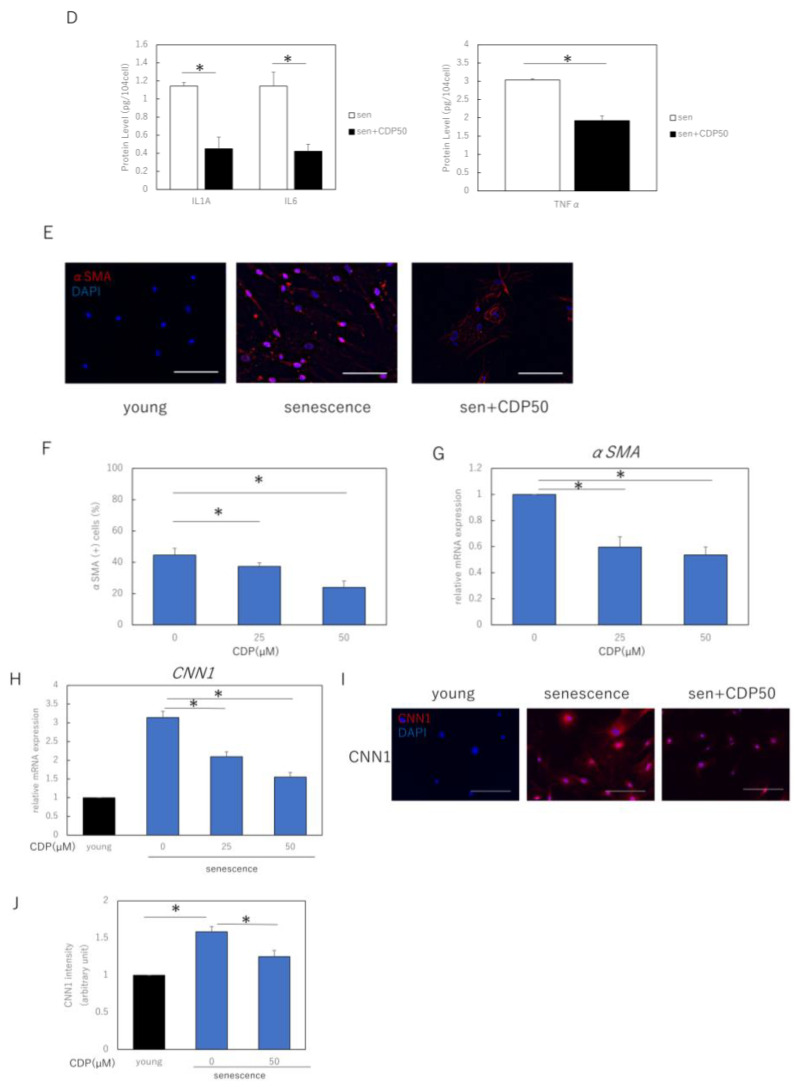
Effects of CDP on the aging phenotype of NHDFs. (**A**) Effect of CDP on SA-β-galactosidase activity. Bar = 100 µm; (**B**) effect of CDP on expression of aging-related protein p16 and longevity protein SIRT1; (**C**) effect of CDP on the expression of SASP-associated genes; (**D**) effect of CDP on the amounts of SASP-related proteins in cell culture medium; (**E**,**F**) changes in αSMA expression induced by CDP treatment (fluorescence immunostaining). Red: αSMA, blue: DAPI (nuclei). Percentage of SMA-positive cells per total cells was calculated from 15 microscopic fields of view in each group; Bar = 100 µm; (**G**) CDP treatment-induced changes in αSMA gene expression; (**H**) expression of CNN1 (reticular marker) in the CDP-treated NHDFs; (**I**) changes in CNN1 expression in the CDP-treated NHDFs (fluorescence immunostaining). Red: αSMA, blue: DAPI (nuclei). Bar = 50 µm. (**J**) Evaluation of fluorescence intensity in the CNN1 positive regions. Calculated from five microscopic fields of view for each group. Bar = 100 µm. * *p* < 0.05.

We evaluated the effects of CDP treatment on the senescent phenotype of NHDFs. The percentage of senescence-associated (SA)-β-galactosidase-positive NDHF was significantly reduced by CDP treatment (Figure 2A). The treatment reduced p16 expression, a typical aging marker protein, and restored SIRT1 expression, which had decreased with aging (Figure 2B). Furthermore, CDP treatment significantly suppressed the expression of IL1A, IL6, MMP3, MMP9, and tumor necrosis factor (TNF)-α, which are the typical SASP factors of aging NHDFs in skin (Figure 2C). In the NHDF culture model, the number of cells with reticular phenotype and SMA-positive myofibroblasts increased with increasing number of NHDF passages. We evaluated the effect of CDP treatment on the percentage of α-SMA-positive cells; CDP treatment reduced the number of α-SMA-positive cells in highly passage-senescent NHDF (Figure 2D,E). Furthermore, CDP treatment suppressed the expression of α-SMA in senescent HDFs even at the mRNA level (Figure 2F).

We also evaluated the effect of CDP treatment on the expression of CNN1, a gene associated with the reticular phenotype of fibroblasts (Figure 2G). CDP treatment downregulated the expression of CNN1 in aging NHDFs. Moreover, the expression of CNN1 was higher in senescent NHDFs than in young NHDFs. Consistent with the mRNA results, CNN1 protein levels were higher in senescent NHDFs than in young NHDFs (Figure 2H). These findings indicate that senescent NHDFs may have acquired reticular properties. CDP treatment reduced CNN1 expression in senescent NHDFs, suggesting that CDP may have prevented the differentiation of senescent NHDFs into reticular fibroblasts.

### 2.3. Role of NRF2/HO-1 Pathway and Extracellular Signal-Regulated Kinase (ERK) in the Anti-Aging Effects of CDP Treatment

**Figure 3:**
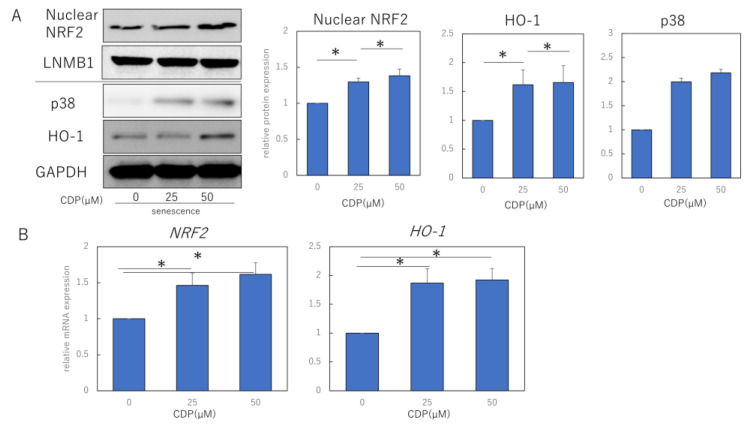

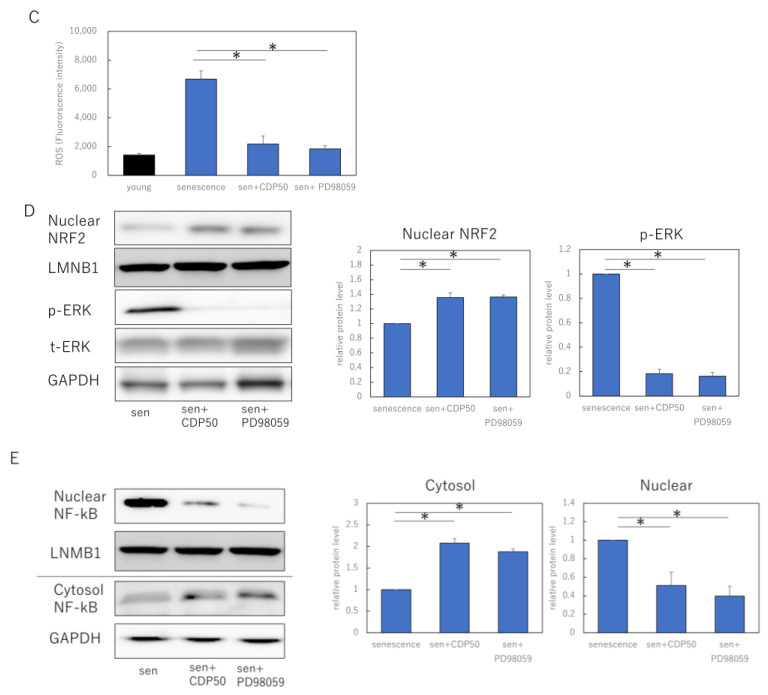
CDP regulates the ROS production by activating the NRF2/HO-1 pathway via suppression of pERK. (**A**) Effect of CDP treatment on p38, nuclear NRF2, and HO-1 expression; (**B**) effect of CDP treatment on NRF2 and HO-1 gene expression by CDP treatment; (**C**) ROS production in NHDFs treated with either CDP or the specific p-ERK1/2 inhibitor, PD98059; (**D**) nuclear NRF2 and ERK1/2 expression in NHDFs treated with either CDP or the specific p-ERK1/2 inhibitor, PD98059; (**E**) expression of each fraction of NF-kB after NHDF treatment with CDP or PD98059. P-ERK, phosphorylated ERK; t-ERK, total ERK; LNMB1; Lamin-B1. * *p* < 0.05.

To clarify the mechanism by which CDP eliminates ROS, we measured the levels of proteins of NRF2/HO-1 antioxidant pathway in NHDFs. The nuclear and cytoplasmic fractions were separated and extracted from cells and subjected to Western blotting analysis. The nuclear fraction was confirmed by expression of LaminB1 as the extracted protein. It was found that p38, nuclear NRF2, and HO-1 protein levels were significantly increased by CDP treatment (Figure 3A). Consistent with the Western blotting results, we observed that mRNA expression levels of NRF2 and HO-1 were also increased by CDP treatment (Figure 3B).

We also examined the effects of CDP on ERK 1/2 signaling, an upstream signal in the NRF/HO-1 pathway. The experiments used 50 μM CDP and the specific p-ERK1/2 inhibitor PD98059; non-CDP-treated senescent HDF cells were characterized by more ROS formation, while ROS production was reduced in CDP- and PD98059-treated cells (Figure 3C). Furthermore, p-ERK1/2 protein expression was significantly suppressed in the CDP- and PD98059-treated cells (Figure 3D). We further assessed the downstream molecules of p-ERK1/2 signaling that are affected by CDP during the aging process and discovered that NF-κB migration from the cytosol to the nucleus was inhibited by CDP in the senescent cells (Figure 3E).

## 3. Discussion

Chronic OS plays a central role in the pathogenesis of many diseases, including age-related inflammation characterized by the expression of SASP inflammatory molecules. The amelioration of age-related SASP inflammation by antioxidant administration is critical. This study demonstrated the potential benefits of CDP in reducing age-related oxidative damage in HDFs. In this study, we evaluated the effects of CDF treatment on aging HDFs and observed that CDF treatment increased the proliferative activity, decreased cell injury, and enhanced NHDF survival under OS. CDF treatment altered aging-associated fibroblast subpopulations and reduced the numbers of myofibroblasts and reticular fibroblasts. CDPs show their anti-aging activity by activating the NRF2/HO-1 antioxidant pathway via suppression of ERK1/2 and the nuclear translocation of NF-kB under OS conditions, which may eliminate intracellular ROS. In addition to regulating ROS production, CDP induces transformational changes in fibroblasts.

Cellular senescence is a persistent state of cell-cycle arrest that occurs after exposure to various stressors. OS is characterized by increased ROS production and is implicated in the pathophysiology of many aging-related diseases, including diabetes, cardiovascular disease, inflammation, cancer, degenerative diseases, ischemia, and anemia [25]. The inflammation is accompanied by increased or excessive ROS production by activated inflammatory and immune cells, which causes further damage and exacerbate OS and the inflammation loop; cytokines, chemokines, and the growth factors released by the ROS-induced signals enhance inflammation [26]. Therefore, inflammation, OS, and aging are closely associated with each other. Therefore, natural substances with antioxidant properties that reduce ROS are potential effective therapeutics for alleviating diseases caused by OS as they act as a shield against free radicals and ROS production [27]. In this study, CDPs reduced the generation of intracellular ROS, suggesting that the mechanism involved the activation of the NRF2/HO-1 antioxidant pathway. Several studies have shown that the Nrf2 signaling cascade plays an important role in inflammatory diseases and OS [28].

In addition, HO-1, an Nrf2 target molecule, degrades heme to produce free iron, which reduces the production of carbon monoxide, biliverdin/bilirubin, and ROS, and its induction is known to improve several inflammatory diseases [29]. The NRF2/HO-1 antioxidant pathway is regulated by the PI3K, NF-κB, and MAPK signaling pathways [30,31]. Although, CDPs have a wide variety of pharmacological properties and different molecular mechanisms have been proposed for their activity, in this study, we showed that CDPs are candidate skin antioxidants with the ability to remove ROS via inhibiting MAPK signaling and subsequent activation of the NRF2/HO-1 pathway. To the best of our knowledge, this is the first study to demonstrate the inhibitory effect of CDP on ROS production and MAPK activation in the skin fibroblasts and report a novel mechanism of action for CDP activity in vitro.

In this study, the activation of the NRF-2/HO-1 pathway by CDP in fibroblasts is considered a hormesis phenomenon. A fundamental feature of physiological hormesis is the stress-induced disruption of dynamic homeostasis by molecular damage and energy depletion, followed by the activation of at least one stress and effector response at the transcriptional, translational, and other physiological levels [32,33]. Among the stress responses (SRs), the activation of the NRF2/HO-1 pathway is considered a primary SR to OS induced by ROS and participation promoters, and cell division and exposure to H_2_O_2_ induced these responses [34]. The fact that CDP treatment led to the nuclear translocation of NRF-2 as an oxidative SR in senescent cells and increased the expression of HO-1, an antioxidant metabolite, reveals a new aspect of CDP as a hormetin in aging biology.

This study has some limitations. First, the effects of CDP were only investigated in in vitro setting, and its effects on animal skin, including that of mice and humans, are unknown. Second, the effects on the aging of other cells that constitute the skin, such as keratinocytes and macrophages, were not investigated. We will address these issues in vivo and conduct additional experiments in different cell lines. Lastly, the effects of long-term administration of CDP on senescent fibroblasts, as well as the changes that occur after exposure to CDP is terminated, should be evaluated in the future for long duration.

Overall, the antioxidant CDP inhibited wound and SASP inflammation and can potentially be used in therapies designed to mitigate aging. Toxic oxidative damage is thought to accelerate the aging process; therefore, reducing oxidative damage is critical for maintaining homeostasis during aging. CDP has multiple bioactive effects, including inhibition of SASP inflammation and aging processes in replicated aging and anti-inflammatory, anti-cancer, and inhibitory effects on melanin biosynthesis. Although this study was an in vitro study and further studies are required to investigate CDP’s effect in humans, our results support the potential of CDP, a naturally occurring compound, as a dietary supplement to promote health during aging.

## 4. Materials and Methods

### 4.1. Reagents

CDP (purity > 98%) was purchased from Solarbio (Beijing, China). Briefly, CDP was extracted by the manufacturer as follows. Approximately 2.0 kg of clean *Cistanche deserticola* was air-dried in an oven at 40 °C and ground to a coarse powder. The powder was extracted in hot ethanol for 3 h. The residue was filtered through gauze to remove the filtrate, diluted with water, refluxed at 90 °C, and centrifuged thrice (1500× *g*) to separate the supernatant. The filtrate was then concentrated under reduced pressure and cooled to room temperature (20–25 °C). Subsequently, 95% ethanol was added, and the mixture was left at 4 °C for 24 h and centrifuged (1500× *g*). After three cycles of water extraction and alcohol precipitation, the precipitate was collected, reconstituted with water, deproteinized, dialyzed, and lyophilized to yield crude CDP. CDP was prepared at stock concentration of 1 mM in phosphate-buffered saline (PBS). The MAPK inhibitor, PD98059, was obtained from Fujifilm Wako Pure Chemical (Osaka, Japan).

### 4.2. Cell Culture

NHDFs were purchased from PromoCell (Heidelberg, Germany) and cultured in 10% heat-inactivated fetal bovine serum, penicillin and streptomycin, Dulbecco’s modified Eagle’s medium (Fujifilm Wako Pure chemical) at 37 °C in a 5% CO_2_ incubator. Replicative senescence was defined as a cell population doubling level (PDL) of ≥50 and no growth for more than 2 weeks. Otherwise, low passages were defined as PDL 10–20. OS induction by H_2_O_2_ was performed by exposing the intermediate passage (PDL 30) HDF cells (80% confluence) to 200 μM H_2_O_2_ (Fujifilm Wako Pure Chemical) for 2 h. Cells were rinsed with PBS, pH 7.4, and then added to fresh complete medium and subjected to analysis and treatment for 48 h after H_2_O_2_ stress. Cell division ability was assessed using the BrdU Cell Proliferation Kit (Sigma-Aldrich, St. Louis, MO, USA) according to the manufacturer’s protocol. Proliferating cells, replicative senescent cells, and H_2_O_2_ OS cells were plated in 96-well plates (5 × 10^3^ cells per well, *n* = 5) and maintained in 100 µL of medium; after 24 h, the cells were treated with the indicated concentrations of either CDP (25, 50 µM) or PD98059 (25 µM) and subjected to analysis after 72 h of incubation. Cell viability was analyzed using the CellTiter-Glo® 2.0 Cell Viability Assay (Promega, Madison, WI, USA). PBS was administered as a negative control and the data were normalized against the control.

### 4.3. SA-β-Galactosidase Assay

SA-β-galactosidase activity in the cells was assessed using the senescence β-galactosidase staining kit according to the manufacturer’s protocol (Cell Signaling Technology, Danvers, MA, USA). 2.5 × 10^4^ cells were seeded in 24-well plate. After 24 h, the growth medium was aspirated, and the wells were washed once with PBS. Fixative solution (1X, 250 μL) was added to each well and the plate was incubated at room temperature (20–25 °C) for 15 min. The wells were then washed twice with PBS, and 1X SA-βGal detection solution (250 µL) was added and incubated at 37 °C overnight. The detection solution was removed and PBS (250 µL) was added, and the plate was gently shaken for 1 h at room temperature (20–25 °C). Bright-field images were collected using an upright microscope (NIKON ECLIPSE Ci-L; Nikon Instruments Inc., Melville, NY, USA) and processed using the ImageJ software (Ver. 1.53; National Institutes of Health, Bethesda, MD, USA).

### 4.4. Measurement of Intracellular ROS and LDH

The cells were cultured in 96-well plates with or without CDP for 72 h using the method described above. To measure intracellular ROS, the working solution of ROS Assay Kit-Highly Sensitive DCFH-DA (Dojindo, Kumamoto, Japan) was added to the medium and incubated at 37 °C for 1 h. Cells were washed with Hanks’ balanced salt solution, and the fluorescence intensity was measured using a SpectraMax Paradigm (Molecular Devices, San Jose, CA, USA) using excitation at: 490–520 nm/emission: 510–540 nm. LDH levels were measured using the lactate dehydrogenase assay kit (Cat no. 299-50601, Dojindo) according to the manufacturer’s protocol.

### 4.5. Immunocytochemistry

After CDP or control treatment, cells were placed on glass slides, fixed in acetone for 5 min at room temperature (20–25 °C), and dried completely before staining. The cells were treated with anti-αSMA (ab5694; 1:100 dilution, Abcam, Cambridge, UK), anti-CNN1 antibody (13938-1-AP, 1:100 dilution, Proteintech, Rosemont, IL, USA), and anti-PDPN antibody (ab 217886; 1:150 dilution, Abcam) and incubated at 4 °C overnight. After washing three times with PBS, the slides were incubated with AlexaFluor555-conjugated anti-rabbit or anti-mouse antibody (Thermo Fisher Scientific, Waltham, MA, USA) diluted 1:2000 in PBS for 1 h at room temperature (20–25 °C). After incubation, the slides were washed three times with PBS and mounted with ProLong Gold Antifade Mountant containing 4′,6-diamidino-2-phenylindole (DAPI) (Thermo Fisher Scientific) to visualize the nuclei.

### 4.6. Protein Analysis

For total protein extraction, cells were lysed in radioimmunoprecipitation assay lysis buffer (Fujifilm Wako Pure Chemical Co., Osaka, Japan) containing protease and phosphatase inhibitors and incubated on ice for 10 min. The lysate was then sonicated for 5 min and centrifuged (800× *g*)at 4 °C for 10 min to remove the insoluble fractions. The nuclear proteins were extracted using the Nuclear/Cytosolic Fractionation Kit (Cosmobio, Tokyo, Japan) according to the manufacturer’s protocol.

Protein lysates (40 µg/well) were separated by sodium dodecyl sulfate-polyacrylamide gel electrophoresis and transferred to polyvinylidene difluoride membranes (Bio-Rad laboratories, Inc., Hercules, CA, USA). After blocking with 3% fat-free milk at room temperature (20–25 °C) for 1 h, the membranes were incubated with the following primary antibodies at 4 °C for 24 h; p16 (ab108349, 1:200 dilution, Abcam), p38 MAPK (#9212, 1:1000 dilution, Cell Signaling Technology), SIRT1 (ab32441, 1:100 dilution, Abcam), glyceraldehyde 3-phosphate dehydrogenase (GAPDH, sc-32233; 1:2000 dilution, Santa Cruz, Santa Cruz, CA, USA), p-ERK (sc-7383, 1:200 dilution, Santa Cruz), p44/42 MAPK (Erk1/2) (#9102, 1:200 dilution, Cell Signaling technology), NF-kB (ab16502, 1:100 dilution, Abcam), Lamin-B1(ab16048, 1:1000, Abcam). The next day, the membranes were incubated with the following secondary antibodies; donkey anti-goat IgG H&L (HRP) (ab6885; 1:5000 dilution, Abcam), goat anti-rabbit IgG H&L (HRP) (ab205718; Abcam), goat anti-mouse IgG H&L (HRP) (ab 205719; 1:5000 dilution, Abcam) at 37 °C for 1 h. After washing, the immunoreactive protein bands were visualized using the electrochemiluminescence detection kit (Immunostar LD, Wako Pure Chemical) for 3–5 min. The bands were imaged using a chemiluminescence imager (ImageQuant LAS4000mini; GE Healthcare, Chicago, IL, USA). Image analysis was performed using the ImageJ software.

### 4.7. Enzyme-Linked Immunosorbent Assay (ELISA)

The levels of IL1a, IL6, and TNF-α in the cell culture medium were determined using the Human IL-1a ELISA Kit (ab178008), Human IL-6 ELISA Kit (ab178013), and Human TNF alpha ELISA Kit (ab181421), respectively, following the manufacturer’s protocol.

### 4.8. RNA Extraction and Reverse Transcription

Total RNA was extracted from the cells using the RNeasy Mini Kit (Qiagen, Hilden, Germany) according to the manufacturer’s instructions. To prepare cDNA, total RNA was mixed with reverse transcriptase, random primers, and deoxynucleotide mixture (Takara Bio, Shiga, Japan), and the mixture was incubated in a T100^TM^ thermal cycler (Bio-Rad Laboratories) at 25 °C for 5 min (annealing), 55 °C for 10 min (synthesis), and 80 °C for 10 min (heat inactivation of reverse transcriptase).

### 4.9. Real-Time Quantitative Polymerase Chain Reaction (RT-qPCR)

RT-qPCR was performed using the Applied Biosystems 7500 Fast Real-Time PCR System (Thermo Fisher Scientific). The PCR was performed in two steps: holding reagents at 95 °C for 3 s (denaturation) and at 60 °C for 30 s (annealing and extension). Forty cycles were performed, and the fluorescence of each sample was measured at the end of every cycle. In the subsequent stage of melting curve analysis, the temperature was increased from 60 °C to 95 °C, and the fluorescence was measured continuously. We evaluated the gene expression of Il-6 (Hs00985639_m1), Il-1a (Hs00174092_m1), MMP9 (Hs00957562_m1), CNN1 (Hs00959434_m1), PDPN (Hs00366766_m1), αSMA (Hs05032285_s1), TNF-α (Hs02621508_s1), NRF2 (Hs01022023_m1), and HO-1 (Hs01110250_m1) (all from Thermo Fisher Scientific) using the PCR master mix (4352042; Applied Biosystems). GAPDH (Hs02786624_g1) was used as a control gene to normalize the gene expression levels. Gene expression levels in the proliferating (young) cell population were used as the baseline, and the fold change values were determined using the 2^−ΔΔCT^ method.

### 4.10. Statistical Analysis

Statistical analyses were performed using SPSS (version 22.0; Chicago, IL, USA), and statistical significance was set at *p <* 0.05. The Mann–Whitney U test was used to determine the statistical significance. One-way ANOVA and Tukey’s post-hoc test were used to compare the differences among three or more groups.

## Data Availability

Data supporting the findings of this study are available from the corresponding author, K. T., upon reasonable request.
